# Comparison of Self-reported Measures of Hearing With an Objective Audiometric Measure in Adults in the English Longitudinal Study of Ageing

**DOI:** 10.1001/jamanetworkopen.2020.15009

**Published:** 2020-08-27

**Authors:** Dialechti Tsimpida, Evangelos Kontopantelis, Darren Ashcroft, Maria Panagioti

**Affiliations:** 1 Institute for Health Policy and Organisation, Centre for Primary Care and Health Services Research, School of Health Sciences, Faculty of Biology, Medicine and Health, The University of Manchester, Manchester, UK; 2 Institute for Health Policy and Organisation, School of Health Sciences, Faculty of Biology, Medicine and Health, The University of Manchester, Manchester, UK; 3 National Institute for Health Research Greater Manchester Patient Safety Translational Research Centre, School of Health Sciences, Faculty of Biology, Medicine and Health, The University of Manchester, Manchester, UK

## Abstract

**Importance:**

An effective and sustainable hearing loss (HL) screening strategy for the early detection of and intervention for HL in older adults is needed.

**Objectives:**

To examine the concordance of self-reported measures of hearing difficulty with objective hearing data and the factors associated with the potential discordances among these measures across different population subgroups of a representative sample of people 50 years and older in England.

**Design, Setting, and Participants:**

This study was a cross-sectional analysis of wave 7 of the English Longitudinal Study of Ageing (ELSA), a large, population-based, prospective cohort study that provides a unique resource for exploring issues associated with aging in England in the 21st century. The full analytic cohort was composed of 9666 individuals participating in the ELSA wave 7, which collected information from June 1, 2014, to May 31, 2015. This study further analyzed a sample of 8529 adults 50 to 89 years of age who had an assessment of their hearing by self-reported measures, and consented to assessment by a qualified nurse via a hearing screening device, and did not have an ear infection or a cochlear implant. Bivariate analyses were performed from July 1 to December 30, 2018, and multivariate analysis from January 1 to June 30, 2019. Multiple logistic regression models examined factors associated with misclassification of hearing difficulties across several categories among those with objectively identified HL.

**Exposures:**

The study examined whether age, marital status, retirement status, indicators of socioeconomic position, and lifestyle factors (such as body mass index, physical activity, and tobacco and alcohol consumption) were associated with the concordance between self-reported hearing problems and manual audiometry among older adults.

**Main Outcomes and Measures:**

Self-reported hearing measures, including hearing in background noise, compared with objective audiometric assessments.

**Results:**

A total of 9666 study participants (5368 female [55.5%]; mean [SD] age, 67.4 [14.4] years) provided responses regarding their hearing difficulties, hearing in noise, quality of care in hearing, and hearing aid recommendation in ELSA wave 7. Within the cohort, 684 individuals (30.2%) with objectively measured HL greater than 35 dB HL at 3.0 kHz went undetected by the self-report measure, whereas the new constructed categories for moderate and moderately severe or severe HL resulted in 9.3% increased sensitivity. Factors associated with misreporting hearing difficulties (while they had objectively measured HL >35 dB HL at 3.0 kHz, in the better-hearing ear) were as follows: female sex (odds ratio [OR], 1.97; 95% CI, 1.18-3.28), no educational qualifications (OR, 1.37; 95% CI, 1.26-2.55), routine or manual occupation (OR, 1.43; 95% CI, 1.28-2.61), tobacco consumption (OR, 1.14; 95% CI, 1.08-1.90), alcohol intake above the low-risk-level guidelines (OR, 1.13; 95% CI, 1.11-2.34), and lack of moderate physical activity (OR, 1.25; 95% CI, 1.03-1.42). Age was largely associated with misreporting of moderately severe to severe HL; the odds were 5.75 (95% CI, 1.17-8.13) higher for those 65 to 74 years of age and 7.08 (95% CI, 1.41-9.30) higher for those 75 to 89 years of age to not report their hearing difficulties compared with those 50 to 64 years of age. In addition, socioeconomic indicators, such as educational level (OR, 1.95; 95% CI, 1.63-6.01) and occupation (OR, 2.07; 95% CI, 1.78-5.40), along with lifestyle factors, such as smoking (OR, 1.46; 95% CI, 1.25-2.48) and alcohol intake above the low-risk-level guidelines (OR, 1.86; 95% CI, 1.67-5.12), were factors associated with misreporting moderately severe or severe HL.

**Conclusions and Relevance:**

The use of a screening measure for audiometric testing and a self-report measure is essential for accurately identifying older people with HL. The results of this study should be considered by HL researchers who analyze self-reported hearing data as a surrogate measurement of audiometric hearing to identify bias in their observed analytic research results.

## Key Points

**Questions:**

Among middled-aged adults, are self-reported measures of hearing concordant with audiometry findings, and is potential discordance associated with age or lifestyle factors?

**Findings:**

In this cross-sectional study of 9666 participants in the English Longitudinal Study of Ageing, self-report measures of hearing had limited accuracy and were not sufficiently sensitive to detect hearing loss. Hearing loss went undetected by the self-reported measures.

**Meaning:**

These findings may inform public health policies relevant to selection of appropriate and validated tools for detecting hearing problems among middle-aged and older adults.

## Teaser

This cross-sectional study examines the concordance of self-reported measures of hearing difficulty with objective hearing data and the factors associated with the potential discordances among these measures in people 50 years and older in England.

## Introduction

Hearing loss (HL) is an important public health concern,^1^ with an estimated 432 million adults worldwide and one-third of people older than 65 years affected by disabling HL.^2^ As a noncommunicable disease, HL is far beyond a sensory disorder and can have profound effects on people’s quality of life,^3,4,5,6^ which reinforces the importance of early detection and intervention for the maintenance of physical and emotional well-being among older adults, where the burden of disease is the highest.^4,7^

The World Health Organization has highlighted the pressing need for measures to promote public health action by facilitating early identification of hearing difficulties that supports prioritization of service provision at the community level and integration within primary care systems.^8^ Large-scale hearing screening programs and tools to detect HL in major health care sectors, such as primary care, do not exist globally, including in high-income countries.^1^ This lack of screening programs^9^ excludes the early detection and treatment of patients with gradually progressive HL,^5^ and the annual cost of unaddressed HL exceeds $750 billion globally.^8^ Moreover, in the absence of HL screening programs that could identify those who are unaware that they have HL (ie, unacknowledged HL),^10^ hearing help-seeking depends on self-recognition of hearing difficulties^9^ as a crucial step for the initiation of contact with a health care professional in primary health care settings^11^ and consequently the referral to ear specialists and hearing aid provision (D.T. et al, unpublished data, 2020).

Self-reported measures are frequently used to gather hearing health data in population-based epidemiologic studies. Evidence indicates a discordance between self-reported and objective measures of hearing because adults self-report HL according to their beliefs, which are influenced by a range of contextual factors.^12,13^ However, the validity and the factors associated with the concordance between self-reported HL and manual audiometry remain mostly unknown.^7^ The hearing measures in the English Longitudinal Study of Ageing (ELSA) are comparable to 7 other global aging surveys with harmonized physical and anthropometric measurements.^14^ Thus, the validation of hearing measures is essential for hearing data quality evaluation and can help explain some of the inconsistencies in findings regarding the association of HL with functional outcomes in older adults.^15,16^ The aims of this study were to examine the concordance of self-reported measures of hearing difficulty in ELSA, with objective hearing data measured by a handheld audiometric screening device, and the factors associated with the potential discordances among these measures across different population subgroups of a representative sample of people 50 years and older in England.

## Methods

### Study Population

We used data from ELSA, which is a large, population-based, prospective cohort study that provides a unique resource for exploring issues associated with aging in England in the 21st century.^17^ The full analytic cohort was composed of 9666 individuals participating in the wave 7 of ELSA, which collected information from June 1, 2014, to May 31, 2015. For the purpose of this cross-sectional analysis, we further analyzed a sample of 8529 adults 50 to 89 years of age who had an assessment of their hearing by self-reported measures, consented for assessment by a qualified nurse via a hearing screening device,^18^ and did not have an ear infection or a cochlear implant. All participants gave written informed consent at the recruitment wave to participate in ELSA and at each subsequent wave. All data were anonymized. Ethical approval was granted by the National Research and Ethics Committee.^19^ This study followed the Strengthening the Reporting of Observational Studies in Epidemiology (STROBE) reporting guideline.

### Outcomes

#### Self-reported Hearing Difficulty

According to ELSA documentation,^19^ hearing difficulty is defined as having declared fair or poor hearing on a 5-point Likert scale (with 1 indicating excellent; 2, very good; 3, good; 4, fair; and 5, poor) or finding it difficult to follow a conversation if there is background noise (such as television, radio, or children playing). The participants who positively answered the last question then answered in a separate question whether they had slight, moderate, or great difficulty in following a conversation if there is background noise. We used that response for a further classification of their hearing difficulty into categories, eliminating those who had indicated slight difficulty following a conversation if there is background noise to allow for a fair comparison with the categories of moderate and moderately severe or severe objectively measured HL (eFigure in the Supplement).

#### Objectively Measured HL

The objective measurement of hearing acuity was performed by the HearCheck Screener (Siemens), a handheld audiometric screening device.^18^ The HearCheck Screener automatically generates 6 tones in total: a fixed series of 3 midfrequency sounds with decreasing volume at 1 kHz (at 55 dB HL, 34 dB HL, and 20 dB HL) and afterwards another 3 pure high-frequency sounds at decreasing intensities at 3 kHz (at 75 dB HL, 55 dB HL, and 35 dB HL), testing for audibility for each sequence and per each ear. Participants indicated when they hear the sound by raising their finger.

The HearCheck Screener is an accurate tool in detecting HL when compared with pure-tone air conduction averages, which are designated as gold standard values. In cases of moderate or worse HL, the HearCheck Screener fulfills all the criteria of high sensitivity, high specificity, and high positive predictive values.^20^

Hearing level was defined as greater than 35 dB HL at 3.0 kHz in the better-hearing ear because this is the level at which intervention for HL is definitely beneficial.^21^ Those with HL were further subdivided according to a categorization that has been previously used in the literature for the characterization of those assessed by the same audiometric screening device^21^ as follows: (1) moderate HL (tones heard at 75 dB HL and 55 dB HL but not at 35 dB HL) or (2) moderately severe or severe HL (tone heard or not at 75 dB HL and tones not heard at 55 dB HL and 35 dB HL).

### Covariates

We selected as indicators of socioeconomic position the highest educational attainment (no qualifications, foreign or other, O level Certificate of Secondary Education, A level [Level 3 Qualification of the National Qualifications Framework], and degree or higher education), tertiles of the self-reported occupation according to the National Statistics socioeconomic classification (routine and manual occupations, intermediate, or managerial and professional), and quintiles of the net household income and the total nonpension wealth (first quintile indicating the lowest and fifth quintile indicating the highest).

We considered as covariates age, sex, and lifestyle factors (such as body mass index, physical activity, and tobacco and alcohol consumption) because these are key risk factors for HL among older adults.^22^ We dichotomized marital status into currently married (married, first and only marriage; in a registered civil partnership; or remarried, in a second or later marriage) or not (single, ie, never married and never registered in a marriage; separated but still legally married; divorced; or widowed). Retirement status was also dichotomized to being currently retired or not.

### Statistical Analysis

Bivariate analyses were performed from July 1 to December 30, 2018, and multivariate analysis from January 1 to June 30, 2019. Descriptive statistical measures were reported on hearing difficulties, hearing in noise, quality of care in hearing, and hearing aid recommendation in ELSA wave 7. Participants’ self-reported and objectively measured HL (moderate and moderately severe or severe) was reported as absolute number (relative frequency). We fitted multiple logistic regression models to identify factors associated with the false-negative report of hearing difficulties in people with objectively identified HL. Age was categorized into 3 groups (50-64, 65-74, and 75-89 years) to allow for a comparison with the study by Benova et al,^9^ which examined the self-reported hearing difficulty in ELSA wave 2. There were no missing values in the hearing data of the final analytical sample, which was specifically chosen for the study to fulfill the criteria of completed assessment of hearing by self-reported measures, with given consent for assessment by pure-tone audiometry and without any ear infection or cochlear implant. Separate analyses were conducted for moderate and moderately severe or severe HL. Because some data were missing at random on many variables, we excluded records with missing data from our analyses, concluding that this would be unlikely to affect the validity of our findings.^23,24^

For all models, odds ratios (ORs) and 95% CIs are presented. The performance of self-reported hearing difficulty with second stage pure-tone audiometry screening (sensitivity, specificity, and positive and negative predictive values as overall test accuracy) was calculated, and the area under the receiver operating characteristic curve represents the accuracy of all models. We used the Hosmer-Lemeshow test as a postestimation tool, which demonstrates the goodness of fit of logistic regression models. A 2-tailed *P* ≤ .05 was considered to be statistically significant. All data were analyzed using Stata, version 14 (StataCorp).

## Results

### Self-Reported Hearing Acuity

A total of 9666 study participants (5368 female [55.5%]; mean [SD] age, 67.4 [14.4] years) provided responses regarding their hearing difficulties, hearing in noise, quality of care in hearing, and hearing aid recommendation in ELSA wave 7^25^ (Figure). Within the cohort, 3801 (39.3%) reported that they had hearing difficulties. Of those 3801 individuals with self-reported hearing difficulty, 1949 (51.3%) did not tell a physician or nurse about their hearing problems, thereby missing the opportunity to be referred for further assessment.

**Figure.**
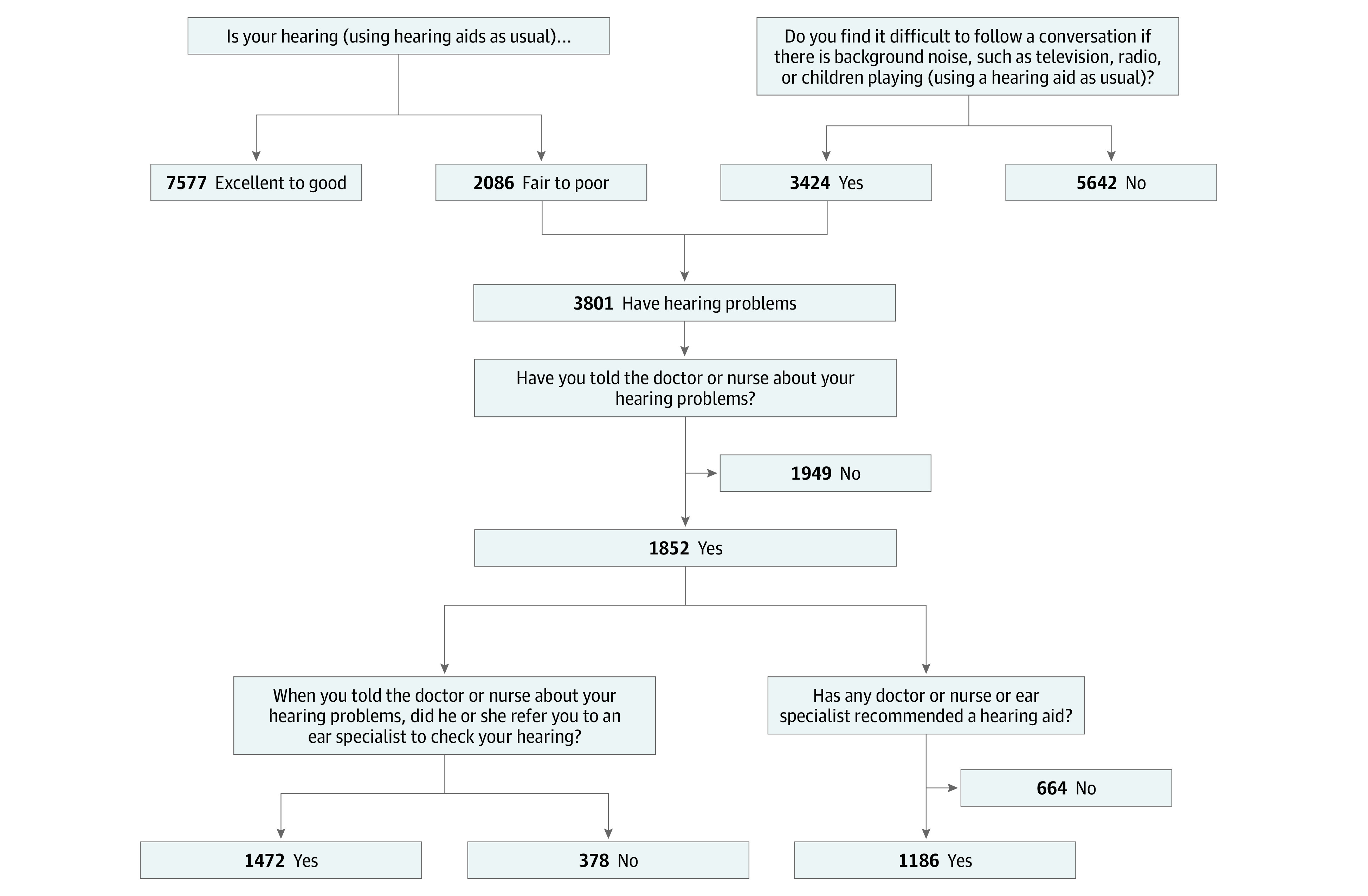
The Questions on Hearing Difficulties, Hearing in Noise, Quality of Care in Hearing, and Hearing Aid Recommendation in the English Longitudinal Study of Ageing Wave 7

Examining the characteristics of the 2 separate categories (not mutually exclusive events) that the self-reported hearing difficulty was composed of, we found that not informing a health care professional was more common among those who reported difficulty in following conversations in the presence of background noise (1753 of 3424 [51.2%]) compared with those who had fair to poor self-reported hearing (691 of 2086 [33.1%]). Importantly, 1894 of the 3425 participants (55.3%) who had reported that they found it difficult to follow a conversation if there is background noise (such as television, radio, or children playing) and did not have hearing aids had reported that they have good, very good, or excellent hearing, which indicates that more than half of them had unacknowledged HL, with 718 of 1894 (37.9%) of them having moderate or great difficulty (Table 1).

**Table 1.** Participant Characteristics on Questions on Hearing Difficulties and Hearing in Noise in ELSA Wave 7

Self-reported hearing	Finds it difficult to follow a conversation if there is background noise, No. (%)^a^				
	Yes (n = 3425)			No (n = 5642)	Total
	Slight difficulty	Moderate difficulty	Great difficulty		
Excellent	86 (5.3)	21 (1.6)	9 (1.9)	1448 (25.7)	17.4 (1678)
Very good	331 (20.5)	119 (8.9)	7 (1.5)	2095 (37.1)	27.7 (2674)
Good	759 (47.1)	478 (35.8)	84 (17.5)	1721 (30.5)	33.4 (3225)
Fair	389 (24.1)	558 (41.8)	185 (38.6)	339 (6.0)	16.3 (1573)
Poor	47 (2.9)	158 (11.8)	194 (40.5)	39 (0.7)	5.3 (513)
Total	1612 (100)	1334 (100)	479 (100)	5642 (100)	9666 (100)

Abbreviation: ELSA, English Longitudinal Study of Ageing.

^a^ Three participants in total answered that they do not know whether they find it difficult to follow a conversation if there is background noise.

Eliminating from the categories of self-reported hearing difficulties those that had indicated slight difficulty following a conversation if there is background noise (such as television, radio, or children playing), we had 2249 complete cases with hearing difficulty instead of the initial 3505. This sample size improved the classification accuracy substantially for those with self-reported hearing difficulty, resulting in 9.3% increased sensitivity (79.1%), which refers to the ability of the self-reported measure to correctly identify those with HL (true-positive results) (Table 2). Under that new categorization, 20.9% of those with HL as measured by the handheld audiometric screening device went undetected by the self-reported measure instead of 30.2% (false-negative results: 298 of 1427).

**Table 2.** Statistical Outcomes of Complete Cases With Self-reported and Objective Hearing Data in ELSA Wave 7

Outcome	Self-reported hearing difficulty (n = 3505)^a^	New categorization of self-reported hearing difficulty (n = 2036)^b^
Objectively measured hearing loss, No.	2266	1427
Total overlap, No.	1582	1129
Sensitivity, % (95% CI)	69.8 (67.9-71.7)	79.1 (76.9-81.2)
Specificity, % (95% CI)	69.3 (68.1-70.4)	47.8 (45.4-50.1)
Positive predictive value, % (95% CI)	45.1 (43.5-46.8)	55.5 (53.3-57.6)
Negative predictive value, % (95% CI)	86.4 (85.4-87.3)	73.5 (70.8-76.1)
Positive likelihood ratio (95% CI)	2.3 (2.2-2.4)	1.5 (1.4-1.6)
Negative likelihood ratio (95% CI)	0.4 (0.4-0.5)	0.4 (0.4-0.5)
ROC area (95% CI)	0.69 (0.68-0.71)	0.64 (0.62-0.65)

Abbreviations: ELSA, English Longitudinal Study of Ageing; ROC, receiver operating characteristic.

^a^ Current categories of self-reported measures are as follows: the sum of those who rated their hearing as fair or poor on a 5-point Likert scale (with 1 indicating excellent; 2, very good; 3, good; 4, fair; and 5, poor) or responded positively to the question of whether they find it difficult to follow a conversation if there is background noise (such as television, radio, or children playing).

^b^ New categorizations of self-reported measures are as follows: the sum of those who rated their hearing as fair or poor on a 5-point Likert scale (with 1 indicating excellent; 2, very good; 3, good; 4, fair; and 5, poor) or responded that they have moderate or great difficulty in following a conversation if there is background noise (such as television, radio, or children playing).

### Objectively Measured HL

Table 3 gives the distribution of sociodemographic characteristics of participants’ self-reported and objectively measured HL. Table 4 gives the summary of multiple logistic regression for variables associated with false-negative report of hearing difficulties on the sample with (1) objectively identified HL greater than 35 dB HL at 3.0 kHz (n = 2266), (2) moderate HL at 3.0 kHz (n = 1498), and (3) moderately severe or severe HL at 3.0 kHz (n = 768) in the better-hearing ear of 8529 participants 50 to 89 years of age in ELSA wave 7.

**Table 3.** Participants’ Self-reported and Objectively Measured HL in the Better-Hearing Ear

Variable	No. (%) of participants (n = 8529)					
	Self-reported measurement			Objective measurement		
	Self-reported hearing difficulty (n = 2249)	Moderate self-reported hearing difficulty (n = 1565)	Moderately severe or severe self-reported hearing difficulty (n = 684)	HL >35 dB HL at 3.0 kHz (n = 2266)	Moderate HL (n = 1498)^a^	Moderately severe or severe HL (n = 768)^b^
Sex						
Male	1243 (55.3)	832 (53.2)	411 (60.1)	1198 (52.9)	741 (49.5)	457 (59.5)
Female	1006 (44.7)	733 (46.8)	273 (39.9)	1068 (47.1)	757 (50.5)	311 (40.5)
Age group, y						
50-64	624 (28.9)	456 (30.2)	168 (26.0)	349 (16.2)	280 (19.3)	69 (9.8)
65-74	739 (34.2)	545 (36.0)	194 (30.0)	722 (33.6)	535 (36.9)	187 (26.7)
75-89	796 (36.9)	511 (33.8)	285 (44.0)	1081 (50.2)	636 (43.8)	445 (63.5)
Missing	90 (4.0)	53 (3.3)	37 (5.4)	114 (5.0)	47 (3.1)	67 (8.7)
Currently married						
No	814 (36.2)	562 (35.9)	252 (36.8)	826 (38.4)	544 (37.5)	282 (40.2)
Yes	1435 (63.8)	(1003 (64.1)	432 (63.2)	1326 (61.6)	907 (62.5)	419 (59.8)
Missing	0	0	0	114 (5.0)	47 (3.1)	67 (8.7)
Retirement status						
Retired	1563 (69.5)	1076 (68.8)	487 (71.2)	1685 (78.3)	1112 (76.6)	573 (81.7)
Not retired	686 (30.5)	489 (31.2)	197 (28.8)	467 (21.7)	339 (23.4)	128 (18.3)
Missing	0	0	0	114 (5.0)	47 (3.1)	67 (8.7)
Educational level						
Degree or higher education	646 (29.5)	471 (31.0)	175 (26.2)	562 (26.5)	404 (28.2)	158 (22.9)
A level	180 (8.2)	127 (8.3)	53 (7.9)	137 (6.5)	100 (6.9)	37 (5.4)
O level CSE	476 (21.7)	350 (23.0)	126 (18.9)	473 (22.3)	321 (22.4)	152 (22.0)
Foreign or other	256 (11.7)	182 (12.0)	74 (11.1)	252 (11.9)	171 (11.9)	81 (11.7)
No qualifications	632 (28.9)	392 (25.8)	240 (35.9)	701 (33.0)	439 (30.6)	262 (38.0)
Missing	59 (2.6)	43 (2.7)	16 (2.3)	141 (6.2)	63 (4.2)	78 (10.1)
Occupation-based National Statistics Socioeconomic Classification	484 (24.8)	353 (26.0)	131 (22.0)	423 (21.5)	285	138 (21.2)
Managerial and professional occupations	684 (35.0)	495 (36.5)	189 (31.7)	665 (33.8)	477	188 (28.9)
Intermediate occupations (nonmanual)	784 (40.2)	508 (37.5)	276 (46.3)	881 (44.7)	556	325 (49.9)
Routine and manual occupations	297 (13.2)	209 (13.3)	88 (12.8)	297 (13.1)	180 (12.0)	117 (15.2)
Net household income						
Fifth quintile (highest)	284 (14.3)	217 (15.7)	67 (11.2)	243 (12.3)	178 (13.4)	65 (10.1)
Fourth quintile	391 (19.7)	291 (21.0)	100 (16.7)	367 (18.6)	265 (19.9)	102 (15.9)
Third quintile	461 (23.2)	297 (21.4)	164 (27.3)	453 (23.0)	297 (22.3)	156 (24.3)
Second quintile	460 (23.2)	312 (22.5)	148 (24.7)	489 (24.8)	329 (24.7)	160 (24.9)
First quintile (lowest)	389 (19.6)	268 (19.4)	121 (20.2)	421 (21.3)	262 (19.7)	159 (24.8)
Missing	264 (11.7)	180 (11.5)	84 (12.2)	293 (12.9)	167 (11.1)	126 (16.4)
Net financial wealth						
Fifth quintile (highest)	386 (19.5)	280 (20.2)	106 (17.7)	342 (17.3)	243 (18.3)	99 (15.4)
Fourth quintile	391 (19.7)	283 (20.4)	108 (18.0)	400 (20.3)	284 (21.3)	116 (18.1)
Third quintile	457 (23.0)	331 (24.0)	126 (21.0)	466 (23.6)	311 (23.4)	155 (24.1)
Second quintile	443 (22.3)	301 (21.7)	142 (23.7)	475 (24.1)	294 (22.1)	181 (28.2)
First quintile (lowest)	308 (15.5)	190 (13.7)	118 (19.6)	290 (14.7)	199 (14.9)	91 (14.2)
Missing	264 (11.7)	180 (11.5)	84 (12.2)	293 (12.9)	167 (11.1)	126 (16.4)

Abbreviations: CSE, Certificate of Secondary Education; HL, hearing loss.

^a^ Moderate HL: tones heard at 75 dB HL and 55 dB HL but not at 35 dB HL (the first 2 of the 3 tones at 3.0 kHz heard).

^b^ Moderately severe or severe HL: tone heard or not at 75 dB HL and tones not heard at 55 dB HL and 35 dB HL (0 or 1 of the 3 tones at 3.0 kHz heard).

**Table 4.** Summary of Multiple Logistic Regression for Variables Associated With False-Negative Report of Hearing Difficulties by Sample^a^

Variable	Model 1^b^	Model 2^c^	Model 3^d^
Female sex	1.97 (1.18-3.28)	0.94 (0.55-1.60)	1.23 (1.18-3.16)
Age of 65-74 y	0.59 (0.28-1.26)	0.86(0.40-1.84)	5.75 (1.17-8.13)
Age of 75-89 y	0.55 (0.25-1.21)	0.85 (0.37-1.94)	7.08 (1.41-9.30)
Retirement status (not retired)	0.92 (0.46-1.78)	1.13 (1.08-2.15)	1.07 (0.39-2.93)
Educational level (no qualifications)	1.37 (1.26-2.55)	1.07 (1.05-2.45)	1.95 (1.63-6.01)
Occupation (routine or manual)	1.43 (1.28-2.61)	1.66 (1.09-1.98)	2.07 (1.78-5.40)
Income (lowest)	0.94 (0.77-1.15)	1.69 (1.19-3.19)	0.97 (0.73-1.27)
Tobacco use (current or former)	1.14 (1.08-1.90)	2.32 (1.80-3.75)	1.46 (1.25-2.48)
Excessive alcohol consumption (>14 units per week)	1.13 (1.11-2.34)	0.99 (0.97-1.02)	1.86 (1.67-5.12)
Physical activity (moderate sports or activities hardly ever or never)	1.25 (1.03-1.42)	1.10 (0.82-1.47)	1.02 (0.73-1.41)
Hosmer-Lemeshow χ^2^	9.43	3.82	11.39
Probability > χ^2^	0.31	0.87	0.18

Abbreviation: HL, hearing loss.

^a^ Data are presented as odds ratio (95% CI) unless otherwise indicated.

^b^ Model 1: did not report hearing difficulties while they had objectively measured HL by HearCheck (>35 dB HL at 3.0 kHz in the better-hearing ear) (n = 2266).

^c^ Model 2: did not report moderate hearing difficulties while they had objectively measured moderate HL (n = 1498); objective moderate HL: tones heard at 75 dB HL and 55 dB HL but not at 35 dB HL (the first 2 of the 3 tones at 3.0 kHz heard).

^d^ Model 3: did not report moderately severe or severe hearing difficulties while they had objectively measured moderately severe or severe HL (n = 768); objective moderately severe or severe HL: tone heard or not at 75 dB HL and tones not heard at 55 dB HL and 35 dB HL (0 or 1 of the 3 tones at 3.0 kHz heard).

The multiple logistic regression models showed that demographic, socioeconomic, and lifestyle factors were associated with the inaccuracy in the self-identification of the objectively identified HL. Significant factors associated with total misreporting were female sex (OR, 1.97; 95% CI, 1.18-3.28), no educational qualifications (OR, 1.37; 95% CI, 1.26-2.55), routine or manual occupation (OR, 1.43; 95% CI, 1.28-2.61), tobacco consumption (OR, 1.14; 95% CI, 1.08-1.90), alcohol intake above the low-risk level guidelines (OR, 1.13; 95% CI, 1.11-2.34), and lack of moderate physical activity (OR, 1.25; 95% CI, 1.03-1.42).

Age was largely associated with misreporting of moderately severe to severe HL; the odds were 5.75 (95% CI, 1.17-8.13) higher for those 65 to 74 years of age and 7.08 (95% CI, 1.41-9.30) for those 75 to 89 years of age to not report their hearing difficulties compared with those 50 to 64 years of age. In addition, socioeconomic indicators, such as education (OR, 1.95; 95% CI, 1.63-6.01) and occupation (OR, 2.07; 95% CI, 1.78-5.40), along with lifestyle factors, such as smoking (OR, 1.46; 95% CI, 1.25-2.48) and alcohol intake above the low-risk level guidelines (OR, 1.86; 95% CI, 1.67-5.12) were factors associated with misreporting moderately severe or severe HL.

## Discussion

In this study, we examined the validity of self-reported measures compared with HL as measured by the HearCheck Screener. We found that in a population-based sample of 8529 adults 50 to 89 years of age, nearly one-third of those had objectively identified HL that went undetected by the self-report measures. These findings suggest that the use of a screening measure for audiometric testing along with a self-report measure in epidemiologic studies and clinical practice is essential for accurately identifying older people with HL. Moreover, we found that female sex, older age, socioeconomic inequalities, and unhealthy lifestyle (tobacco use, alcohol intake above the low-risk level guidelines, and lower levels of physical activity), which are recognized as key risk factors for HL among older adults,^22^ were largely associated with the inaccuracy of self-identification of hearing difficulties in those with objectively identified HL.

### Comparison With Previous Literature

Our findings are consistent with previous studies^7,10,15,26,27,28,29,30,31^ that have examined on a smaller scale the performance of self-reported hearing difficulties in combination with pure-tone audiometry among elderly individuals. However, to our knowledge, our study is the first vigorous examination of the validity of self-reported measures of hearing, including difficulties in background noise, with objective audiometric assessments in such a large and nationally representative cohort.

In general, all studies except for the studies by Diao et al^26^ and Ferrite et al^31^ argued that self-reported hearing should not be considered representative in associations with functional outcomes. The study by Diao et al^26^ concluded that the Hearing Handicap Inventory for the Elderly-Screening Version (HHIE-S) could be considered a reliable and valid screening tool. Professional organizations have suggested the use of HHIE-S in combination with pure-tone screening because HHIE-S is focused mainly on the assessment of the social and emotional aspects of HL on the individual (handicap) and not the self-reported hearing ability.^26^ The study by Ferrite et al^31^ focused on a small sample (n = 188) of a younger adult population (30-65 years of age) drawn from a population-based cohort study, which may reveal that different factors may affect the sensitivity and specificity of self-reported hearing measures in an older population.

The role of age and sex in the inaccuracy of the self-reported measures has also been highlighted by Kamil et al,^28^ who found that the agreement rates between subjective and objective hearing measures were lower among the older age group (≥60 years of age) and among women. This finding may reflect that people tend to undervalue the importance of hearing and consider its loss as an inevitable accompaniment of getting older^22^ and therefore adapt to HL over time,^16^ underestimating the magnitude of their HL.^29^

Regarding the role of socioeconomic position, our findings are consistent with previous studies that found that agreement rates between subjective and objective hearing measures were relatively lower among those of a lower educational attainment^28^ and occupational groups subject to noise-induced HL.^32^ The role of income in the false-negative report of hearing difficulties may reflect financial barriers to the use of and access to hearing health care^33^ and the downgrade of HL as a health priority.^26^

### Implications for Research, Policy, and Practice

These findings have important public health implications and call for a revised assessment approach for HL in older adults; clinical research often relies on self-report measure of HL, but our findings indicate that this could not be regarded as a well-suited and accurate measure to identifying individuals with HL without the additional use of a screening measure for audiometric testing.^27^ The underestimation of hearing difficulties poses a significant barrier to HL intervention, and the self-report measures should not be considered reliable measures of hearing acuity to influence the judgment for referral to secondary care.

The help-seeking behavior for hearing difficulties starts with individuals’ self-diagnosis and initiation of contact with a health care professional in primary health care settings.^13^ In addition, unacknowledged HL constitutes a significant nonfinancial barrier. The existence of objective hearing measures is crucial, particularly for those belonging to high-risk groups that are most likely to remain unrecognized, such as people who face socioeconomic inequalities and adopt an unhealthy lifestyle, because these factors may affect the initiation of help-seeking and consequently the referral to ear specialists. Our findings address important conflicts in the literature, shedding light on the inconsistencies across studies regarding the association of HL with functional outcomes^15^ and may reflect attitudinal differences across different cultures and geographic variation in the acknowledgment of hearing difficulties.

### Strengths and Limitations

The main strength of our study is that it provides the largest and most accurate evaluation of the discordance between objective and self-reported measures of HL today. Our study is also the first, to our knowledge, to address the association of lifestyle factors with the agreement rate, which had not been previously examined in the literature.^15^ However, the study also has significant limitations. First, the cross-sectional analyses did not allow for causal or temporal relationships among the factors associated with the inaccuracy of self-reported measures. In addition, questionnaires that contain few questions to assess hearing deficits may have validity.^34^ A relatively small proportion of participants who responded having good, very good, or excellent hearing were also using a hearing aid, which may have confounded their response. Finally, the comparison of self-reported measure to the results from the HearCheck Screener may contain information bias because the screening tool identified only those with HL greater than 35 dB HL at 3.0 kHz in the better-hearing ear, whereas the self-reported questions did not specify that criterion.

## Conclusions

Our study found that self-report measurement of HL had limited concordance with objective measures of HL. In light of these findings, the importance of an effective and sustainable HL screening strategy for the early detection and intervention for HL in older adults is reinforced. The lack of screening programs excludes the early detection and treatment of patients with gradually progressive HL, especially those with unacknowledged HL. These results should be considered by HL researchers who analyze self-reported hearing data as a surrogate measurement of audiometric hearing to identify bias in their observed analytic research results. Future research should examine the role of other environmental and personal factors in the agreement rate between self-reported and objective measures of hearing, for which little is known,^15^ and investigate sociospatial hearing health inequalities.

## Notes

Supplement. **eFigure.** Definitions of Categories of Self-Reported Hearing Difficulty
