# Supplementary material for: Comparison of Self-reported Measures of Hearing With an Objective Audiometric Measure in Adults in the English Longitudinal Study of Ageing
Source: JAMA Netw Open. 2020 Aug 27;3(8):e2015009. doi: 10.1001/jamanetworkopen.2020.15009 (PMC7453309; doi:10.1001/jamanetworkopen.2020.15009)
Supplement: Supplement. — eFigure. Definitions of Categories of Self-Reported Hearing Difficulty [file jamanetwopen-3-e2015009-s001.pdf]

Supplementary Online Content

Tsimpida D, Kontopantelis E, Ashcroft D, Panagioti M. Comparison of self-reported measures of hearing with an objective audiometric measure in adults in the English Longitudinal Study of Ageing. *JAMA Netw Open*. 2020;3(8):e2015009.
doi:10.1001/jamanetworkopen.2020.15009

eFigure. Definitions of Categories of Self-reported Hearing Difficulty

This supplementary material has been provided by the authors to give readers additional information about their work.

© 2020 Tsimpida D et al. *JAMA Network Open.*

eFigure. Definitions of Categories of Self-reported Hearing Difficulty

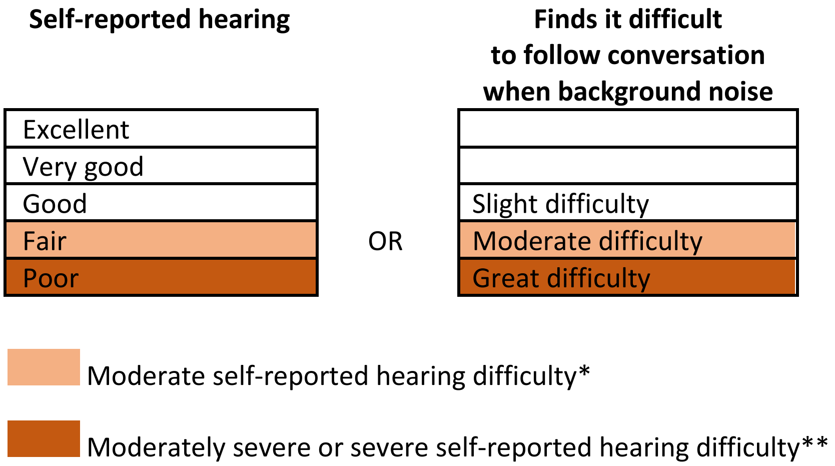

* Moderate self-reported hearing difficulty: if hearing was *fair* OR had *moderate* difficulty following a conversation in background noise.
** Moderately severe or severe self-reported hearing difficulty: if hearing was *poor* OR had *great difficulty* following a conversation in background noise.

© 2020 Tsimpida D et al. *JAMA Network Open.*
